# Aspersymmetide A, a New Centrosymmetric Cyclohexapeptide from the Marine-Derived Fungus *Aspergillus versicolor*

**DOI:** 10.3390/md15110363

**Published:** 2017-11-22

**Authors:** Xue-Mei Hou, Ya-Hui Zhang, Yang Hai, Ji-Yong Zheng, Yu-Cheng Gu, Chang-Yun Wang, Chang-Lun Shao

**Affiliations:** 1 Key Laboratory of Marine Drugs, The Ministry of Education of China, School of Medicine and Pharmacy, Ocean University of China, Qingdao 266003, China; houxuemei_1990@163.com (X.-M.H.); 15689932652@163.com (Y.-H.Z.); haiyangom@163.com (Y.H.); 2 Laboratory for Marine Drugs and Bioproducts, Qingdao National Laboratory for Marine Science and Technology, Qingdao 266200, China; 3 State Key Laboratory for Marine Corrosion and Protection, Luoyang Ship Material Research (LSMRI), Qingdao 266061, China; zhengjy@sunrui.net; 4 Syngenta, Jealott’s Hill International Research Centre, Bracknell RG42 6EY, Berkshire, UK; yucheng.gu@syngenta.com; 5 Institute of Evolution & Marine Biodiversity, Ocean University of China, Qingdao 266003, China

**Keywords:** gorgonian-derived fungus, *Carijoa* sp., *Aspergillus versicolor*, centrosymmetric cyclohexapeptide, cytotoxicity

## Abstract

A new centrosymmetric cyclohexapeptide, aspersymmetide A (**1**), together with a known peptide, asperphenamate (**2**), was isolated from the fungus *Aspergillus versicolor* isolated from a gorgonian coral *Carijoa* sp., collected from the South China Sea. The chemical structure of **1** was elucidated by analyzing its NMR spectroscopy and MS spectrometry data, and the absolute configurations of the amino acids of **1** were determined by Marfey’s method and UPLC-MS analysis of the hydrolysate. Aspersymmetide A (**1**) represents the first example of marine-derived centrosymmetric cyclohexapeptide. Moreover, **1** exhibited weak cytotoxicity against NCI-H292 and A431 cell lines at the concentration of 10 μM.

## 1. Introduction

Marine-derived peptides with a hybrid biosynthetic pathway, non-ribosomal peptide synthetases (NRPSs) and polyketide synthetases (PKSs), always show diverse chemicals and activities [1,2,3]. These peptides provide many prophylactic and curative medicinal drugs with wide bioactivities such as antimalarial, antitumor, antimicrobial, antiviral, and cardioprotective actions [4,5,6]. Efforts by many research groups focusing on hybrid peptides from marine organisms have been rewarded by the discoveries of novel and bioactive compounds, and some of them have been clinically studied and approved by FDA for disease treatments. For example, the linear peptides E7974 [7], ASG-5ME [8], SGN-75 [9], and CDX-011 [10], cyclic peptide elisidepsin [11], and aplidine [12] are anticancer agents within Stages I–III clinical trials. Besides, SGN-35 [13] and ziconotide [14,15], semi-synthetic and natural peptides for the treatment of cancer and pain, respectively, have been approved by FDA. It has been illustrated that marine-derived peptides with NRPS/PKS biosynthetic pathway may have the great potential as lead compounds for drug development.

**Figure 1:**
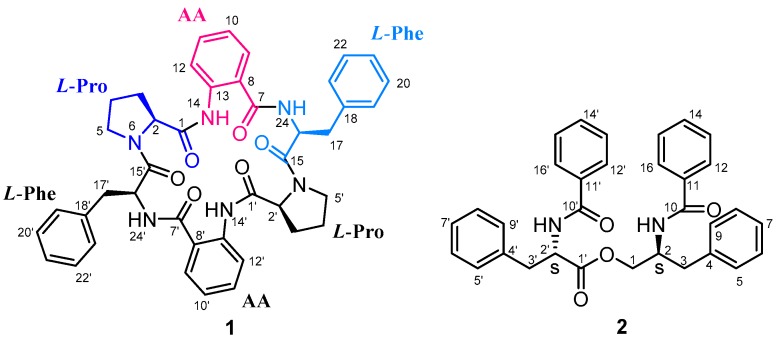
Chemical structures of **1** and **2**.

During our research on bioactive metabolites of marine organisms collected from the South China Sea, several bioactive peptides have been isolated from the fungi derived from corals [16,17,18,19]. Recently, chemical investigation of the culture of marine-derived fungus *Aspergillus versicolor* (TA01-14) isolated from a gorgonian *Carijoa* sp. resulted in the isolation of a new centrally symmetrical NRPS/PKS-derived cyclohexapeptide, aspersymmetide A (**1**), and a known peptide, asperphenamate (**2**) [20] (Figure 1). Herein, we report the isolation, structure elucidation, and biological evaluation of these compounds.

## 2. Results and Discussion

Aspersymmetide A (**1**) was obtained as a white powder. (+)-HRESIMS of **1** gave [M + H]^+^ and [M + Na]^+^ at *m*/*z* 727.3245 and 749.3062, respectively, indicating a molecular formula of C_42_H_42_N_6_O_6_ with 25 degrees of unsaturation. The ^1^H NMR spectrum (Table 1) exhibited two amide (NH) protons at *δ*_H_ 12.23 and 9.10, two *α*-protons of amino acids at *δ*_H_ 5.24 and 4.47, one 1,2-disubstituted benzene ring and one mono-substituted benzene ring (*δ*_H_ 6.83–7.89). The ^13^C NMR spectrum revealed the presence of three amide carbonyls at *δ*_C_ 169.8, 168.4, and 167.1, twelve aromatic carbons at *δ*_C_ 140.2, 138.3, 132.0, 129.6 (2C), 127.6 (2C), 127.5, 125.8. 121.2, 117.9, and 115.1, and three nitrogen-bearing carbons at *δ*_C_ 60.5, 51.6, and 46.7.

**Figure 2:**
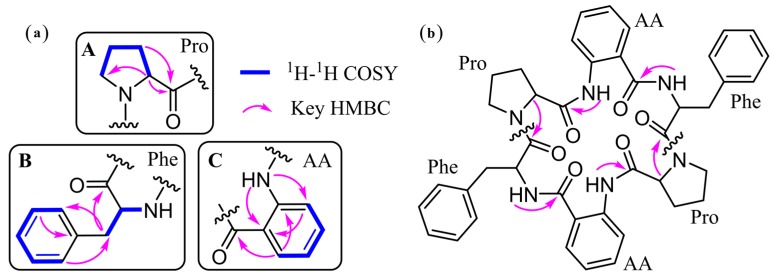
^1^H-^1^H COSY and key HMBC correlations of **1**. (**a**) Residues in **1**. (**b**) The connection of the residues.

**Figure 3:**
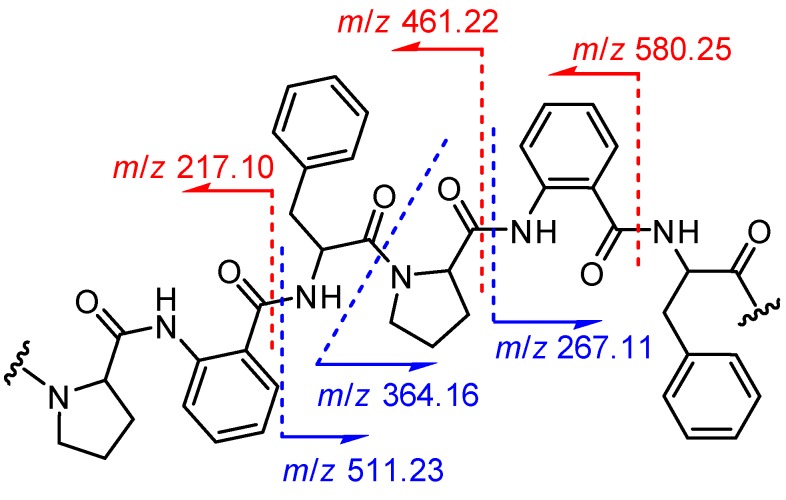
ESI MS^2^ fragment ions for **1**.

Detailed analysis of 1D and 2D NMR data led to the identification of three units including a proline (Pro.), a phenylalanine (Phe.), and an anthranilic acid (AA) (Figure 2a). However, these three units only were attributed to be half of the proposed molecular formula. It suggested that **1** should be a symmetrical dimer. The HMBC correlations were used to connect the residues in **1**. The correlations [Phe-NH→AA-CO] and [AA-NH→Pro-CO] revealed the half sequence of CO-Phe-NH→CO-AA-NH→CO-Pro-N (Figure 2). The [Pro-α-H→Phe-CO] connected the two half sequences to establish the whole cyclic structure, cyclo-[CO-Phe–AA–Pro–Phe–AA–Pro-N]. Additional evidence confirmed the structure of **1** on the basis of the ESI MS^2^ experiments with neutral losses (Figure 3 and Figure S14). Thus, the planar structure of **1** was determined as shown in Figure 2b.

The absolute configurations of the amino acids in **1** were determined by UPLC-MS analysis of the acid hydrolysate derivatized with Marfey’s reagent (*N*^α^-(2,4-dinitro-5-fluorophenyl)-l-alalinamide, l-FDAA) [21]. The retention times and negative ESIMS indicated the presence of l-Pro and l-Phe in **1** (see Experimental and Figure S16). Thus, the absolute configuration of **1** was determined as shown in Figure 1. These structural features revealed that aspersymmetide A (**1**) is a centrally symmetric cyclohexapeptide.

Centrosymmetric cyclopeptides (CSCs) are an important class of peptides that always show diverse bioactivities, such as the enniatins [22] with antibiotic, antifungal, antiinsectan, and cytotoxic activities, and PF1022 [23] with anthelmintic activity. Particularly, fusafungine [24], a mixture of enniatins, has been an active agent used in antibiotics for treatment of nasal and throat infection; emodepdide, the bis-para morpholino-PF1022A, has been introduced into the market as a broad spectrum anthelmintic [25]. A literature survey revealed that the majority of CSCs are synthetic [26,27,28,29], while only a few have been found in natural sources, including cyclohexadepsipeptides (**3**–**10**) [22,30,31,32,33,34,35,36,37,38,39,40] and cyclooctadepsipeptides (**11**–**14**) [25,34,41,42,43,44] (Table 2, Figure S17). Compounds **3**–**13** were obtained from terrestrial microorganisms, and **14** was isolated from the marine-derived bacterium *Micromonospora* sp. aspersymmetide A (**1**) represents the first example of centrally symmetric cyclohexapeptide from marine organisms.

Aspersymmetide A (**1**) was evaluated for brine shrimp lethality against *Artemia salina*, for cytotoxicity against the human breast cancer (MCF-7), human pulmonary carcinoma (NCI-H292), and human skin squamous carcinoma (A-431) cell lines, for antibacterial activity against *Staphylococcus albus* and *Escherichia coli*, for antiviral activity against the human cytomegalovirus (HCMV) and herpes simplex virus (HSV-1), and for enzymic inhibition toward acetyl cholinesterase (AChE), Top I, and *α*-glucosacharase. It displayed weak cytotoxicity against NCI-H292 and A431 cells with an inhibition ratio of 53.84% and 63.62% at a concentration of 10 μM (adriamycin, 1 μM, 93.36% and 91.00%). However, **1** was inactive in other bioassays.

## 3. Materials and Methods

### 3.1. General Experimental Procedures

Optical rotations were measured on a JASCO P-1020 digital polarimeter (JASCO Ltd., Tokyo, Japan). IR spectra were recorded on a Nicolet-Nexus-470 spectrometer (Perkin Elmer Ltd., Boston, MA, USA) using KBr pellets. NMR spectra were recorded on a JEOL JEM-ECP NMR spectrometer (JEOL Ltd., Tokyo, Japan; 500 MHz for ^1^H and 125 MHz for ^13^C), using TMS as internal standard. The ESIMS spectra were obtained from a Micromass Q-TOF spectrometer (Waters Ltd., Boston, MA, USA). Semi-preparative HPLC was performed on a Hitachi L-2000 system (Hitachi Ltd., Tokyo, Japan) using a C18 column (Kromasil (Eka Ltd., Bohus, Sweden) 250 × 10 mm, 5 μm, 2.0 mL/min). UPLC-MS was performed on Waters UPLC^®^ system (Waters Ltd., Boston, MA, USA) using a C18 column (ACQUITY UPLC^®^ (Waters Ltd., Boston, MA, USA) BEH C18, 2.1 × 50 mm, 1.7 μm; 0.5 mL/min) and ACQUITY QDa ESIMS scan from 150 to 1000 Da. Silica gel (Qingdao Haiyang Chemical Group Co., Qingdao, China; 200–300 mesh), octadecylsilyl silica gel (YMC Co., Ltd., Tokyo, Japan; 45–60 μm), and Sephadex LH-20 (GE Ltd., Hartford, CT, USA) were used for column chromatography (CC). Precoated silica gel plates (Yantai Zhifu Chemical Group Co., Yantai, China; G60, F-254) were used for thin layer chromatography.

### 3.2. Fungal Material

The fungus *Aspergillus versicolor* (TA01-14) was isolated from a gorgonian *Carijoa* sp. (GX-WZ-2010001) collected from the Weizhou coral reefs in the South China Sea in April 2010. The strain was deposited at the Key Laboratory of Marine Drugs, the Ministry of Education of China, School of Medicine and Pharmacy, Ocean University of China, Qingdao, China, with the Genbank (NCBI) accession number KP759287.

### 3.3. Fermentation, Extraction, and Isolation

The fungus was cultured on rice solid medium (fifty 1000 mL Erlenmeyer flasks, each containing 50 g of rice and 50 mL of sea water) at room temperature. After 60 days of cultivation, the fermented rice substrate was extracted three times with ethyl acetate (EtOAc) (200 mL per flask) to give an organic extract (10 g). The extract was subjected to a silica gel column chromatography (CC) and eluted by a gradient of petroleum ether (PE)–EtOAc (PE, 100%–0), EtOAc–MeOH (*v:v*, 9:1), and then MeOH to afford eight fractions (Fr.1–Fr.8) on the basis of TLC analysis. Fr.5 was applied over CC of silica gel with PE–EtOAc (PE, 70%–0) to afford three sub-fractions (Fr.5-1–Fr.5-3). Fr.5-3 was then subjected to Sephadex LH-20 CC and eluted with a mixture of CH_2_Cl_2_–MeOH (*v:v*, 1:1) to obtain two sub-fractions (Fr.5-3-1–Fr.5-3-2). Fr.5-3-1 was then repeatedly separated by silica gel and ODS column chromatography, and then purified by HPLC (MeOH–H_2_O, 75–25) to afford Compounds **1** (3 mg) and **2** (2 mg).

Aspersymmetide A (**1**): white powder; ${\left[\alpha \right]}_{D}^{24}$ −174.2 (*c* 0.80, MeOH); UV (MeOH) *λ*_max_ (log *ε*) 206 (4.01), 227 (3.90), 271 (3.34) nm; IR (KBr) ν_max_ 3441, 1632, and 1399 cm^−1^; ^1^H and ^13^C NMR see Table 1; ESI MS^2^ (fragmentation of *m*/*z* 727.52 [M + H]^+^) *m*/*z* 580.25 [M − Phe + H]^+^, 511.23 [M − Pro − AA + H]^+^, 461.22 [M − Phe − AA + H]^+^, 364.16 [M − Pro − AA − Phe + H]^+^, 267.11 [M − Pro − AA − Phe − Pro + H]^+^, 217.10 [M − Phe − AA − Pro − Phe + H]^+^; HRESIMS *m*/*z* 727.3245 [M + H]^+^, 749.3062 [M + Na]^+^ (calcd. for C_42_H_43_N_6_O_6_, 727.3239 [M + H]^+^, C_42_H_42_N_6_O_6_Na, 749.3058 [M + Na]^+^).

The structure of **2** was assigned by spectroscopic method and comparison of the ^1^H- and ^13^C-NMR data (see Supplementary Information) with those reported in the literature [20].

### 3.4. Acid Hydrolysis and Marfey’s Analysis of ***1***

A solution of **1** (0.5 mg) with HCl (6 M, 1 mL) was hydrolyzed by heating for 20 h at 110 °C. The solution was evaporated to dryness under vacuum and redissolved in H_2_O (250 μL). The acid hydrolysate solution (50 μL) was treated with 1% solution of l-FDAA (20 μL) in acetone followed by a solution of NaHCO_3_ (1 M, 10 μL). The mixture was heated at 40 °C for 1 h. The reaction was stopped by HCl (2 M, 5 μL). The standards of amino acids (l-Pro, l/d-Pro, l-Phe, and l/d-Phe) were derivatized with l-FDAA in the same manner as that of **1**. All l-FDAA derivatives were analyzed and detected by UPLCMS (ACQUITY UPLC^®^ (Waters Ltd., Boston, MA, USA)) BEH C18, 2.1 × 50 mm, 1.7 μm; solvents: MeCN (A), H_2_O (0.1% HCOOH) (B); linear gradient: 0–13 min, 5–50% A; 13–15 min, 50–100% A; 15–17 min, 100% A; 17–18 min, 100–5% A; 18–20 min, 5% A; flow rate: 0.5 mL/min; monitor: 190–700 nm; ESI MS scan: 150–1000 Da). Retention times (min) and ESI MS of the amino acid derivatives were recorded as follows: l-FDAA–l-Pro 6.24 min, l-FDAA–d-Pro 6.81 min (*m*/*z* 367.1 [M − H]^−^), l-FDAA–l-Phe 8.92 min, l-FDAA–d-Phe 9.99 min (*m*/*z* 417.1 [M − H]^−^) (Figure S14).

### 3.5. Biological Assay

Brine shrimp lethality against *Artemia salina* was evaluated using the modified Reed-Muench method [45], with doxorubicin as a positive control [46]. Cytotoxic activity was evaluated against the MCF-7, NCI-H292, and A-431 cell lines by the MTT method [47], with adriamycin as a positive control. Antibacterial activity against *S. albus* and *E. coli* was evaluated by using 96-well microtiter plates [48], with ciprofloxacin as a positive control. Antiviral activity against HCMV and HSV-1 was evaluated by the cytopathic effect (CPE) inhibition assay by the MTT method [47], with cidofovir and acyclovir as positive controls, respectively. AChE inhibition was determined spectrophotometrically using acetylthiocholine iodide (ATCI) as substrate by modified Ellman method [49], with huperzine A and galantamine hydrobromide as positive controls. Top I inhibiting activity was tested on the basis of DNA relaxation experiment [50], with 10-hydroxy camptothecin (OPT) as a positive control. *α*-Glucosacharase inhibiting activity was evaluated by the Dewi’s method [51], with acarbose as a positive control.

## 4. Conclusions

A new centrosymmetric cyclopeptide, aspersymmetide A (**1**), was obtained from the gorgonian-derived fungal strain *Aspergillus versicolor* (TA01-14). Compound **1** represents the first example of marine-derived centrosymmetric cyclohexapeptide with weak cytotoxicity.

## Figures and Tables

**Table 1:** ^1^H and ^13^C NMR (500 and 125 MHz, DMSO-*d*_6_) of aspersymmetide A (**1**).

Position		*δ*_H_, Mult. (*J* in Hz)	*δ*_C_, Type
Pro.	1		169.8, C
	2	4.47, d (8.0)	60.5, CH
	3	Ha 2.21, m Hb 1.72, m	31.4, CH_2_
	4	Ha 1.86, m Hb 1.68, m	20.9, CH_2_
	5	Ha 3.52, dd (11.5, 8.5) Hb 3.40, dd (11.5, 10.2)	46.7, CH_2_
AA	7		167.1, C
	8		115.1, C
	9	7.89, d (8.0)	127.5, CH
	10	6.83, t (8.0)	121.2, CH
	11	6.91, t (8.0)	132.0, CH
	12	7.74, d (8.0)	117.9, CH
	13		140.2, C
	14 (NH)	12.23, br s	
Phe.	15		168.4, C
	16	5.24, m	51.6, CH
	17	Ha 3.21, dd (13.6, 5.1) Hb 2.87, dd (13.6, 9.5)	37.2, CH_2_
	18		138.3, C
	19/23	7.19, d (7.3)	129.6, CH
	20/22	7.10, t (7.3)	127.6, CH
	21	7.03, t (7.3)	125.8, CH
	24 (NH)	9.10, d (9.0)	

**Table 2:** Natural products of centrosymmetric cyclopeptides (CSCs).

Compd.	Collected Source	Biosynthetic Source	Bioactivity	Reference
Enniatin A (**3**)	Fungus	*Fusarium* sp.	Anti-mycotoxigenic fungi	[22,30,31]
Enniatin B (**4**)		*Verticillium* sp.		[22,32]
Enniatin C (**5**)		*Verticillium* sp.		[22,32]
Enniatin MK1688 (**6**)		*Fusarium oxysporum*		[22,33]
Verticilide B1 (**7**)		*Verticillium* sp.	Acyl-CoA:cholesterol acyltransferase inhibition	[34]
Himastatin (**8**)	Actinomycete	*Streptomyces hygroscopicus*	Cytotoxic activity	[35,36,37]
Beauvericin (**9**)	Fungus	*Fusarium oxysporum*	Cytotoxic and antiangiogenic activities	[38]
Hirsutellide A (**10**)		*Hirsutella kobayasii*	Antibacterial and antimalarial activities	[39]
Verticilide A1 (**11**)		*Verticillium* sp.	Acyl-CoA:cholesterol acyltransferase inhibition	[34]
Bassianolide (**12**)		*Beauveria bassiana*	Insecticidal activity	[41,42,43]
PF1022A (**13**)		*Ascaridia galli*	Anthelmintic activity	[25]
Thiocoraline (**14**)	Actinomycete	*Micromonospora* sp.	Cytotoxic and antimicrobial activities	[44]

## Supplementary Materials

Supplementary materials according to this paper are available online at www.mdpi.com/1660-3397/15/11/363/s1.
