# Supplementary material for: Aspersymmetide A, a New Centrosymmetric Cyclohexapeptide from the Marine-Derived Fungus Aspergillus versicolor
Source: Mar Drugs. 2017 Nov 22;15(11):363. doi: 10.3390/md15110363 (PMC5706052; doi:10.3390/md15110363)
Supplement: Supplementary file 1 [file marinedrugs-15-00363-s001.pdf]

Aspersymmetide A, a New Centrosymmetric Cyclohexapeptide from the Marine-derived Fungus *Aspergillus versicolor*

Xue-Mei Hou [1,2,3], Ya-Hui Zhang [1,2], Yang Hai [1,2], Ji-Yong Zheng [3], Yu-Cheng Gu [4], Chang-Yun Wang [1,2,5]* and Chang-Lun Shao [1,2,3]*

[1] Key Laboratory of Marine Drugs, The Ministry of Education of China, School of Medicine and Pharmacy, Ocean University of China, Qingdao 266003, The People's Republic of China; houxuemei_1990@163.com (X.-M. H.); 15689932652@163.com (Y.-H. Z.); haiyangom@163.com (Y. H.)

[2] Laboratory for Marine Drugs and Bioproducts, Qingdao National Laboratory for Marine Science and Technology, Qingdao 266200, The People's Republic of China

[3] State Key Laboratory for Marine Corrosion and Protection, Luoyang Ship Material Research (LSMRI), Qingdao 266061, The People's Republic of China; zhengjy@sunrui.net (J.-Y. Z.)

[4] Syngenta, Jealott's Hill International Research Centre, Bracknell, Berkshire, RG42 6EY, United Kingdom; yucheng.gu@syngenta.com (Y.-C. G)

[5] Institute of Evolution & Marine Biodiversity, Ocean University of China, Qingdao 266003, The People's Republic of China

* Correspondence: changyun@ouc.edu.cn (C.-Y. W.), shaochanglun@163.com (C.-L. S.); Tel.: 86-532-82031536 (C.-Y. W.), 86-532-82031381 (C.-L. S.)

Supplementary Information

Figure S1. ^{1}H NMR (500 MHz, DMSO-*d*$_6$) spectrum of compound **1**.

Figure S2. Partial ^{1}H NMR (500 MHz, DMSO-*d*$_6$) spectrum of compound **1**.

Figure S3. Partial ^{1}H NMR (500 MHz, DMSO-*d*$_6$) spectrum of compound **1**.

Figure S4. ^{13}C NMR (125 MHz, DMSO-*d*$_6$) spectrum of compound **1**.

Figure S5. Partial ^{13}C NMR (125 MHz, DMSO-*d*$_6$) spectrum of compound **1**.

Figure S6. HSQC (DMSO-*d*$_6$) spectrum of compound **1**.

Figure S7. ^{1}H–^{1}H COSY (DMSO-*d*$_6$) spectrum of compound **1**.

Figure S8. Partial ^{1}H–^{1}H COSY (DMSO-*d*$_6$) spectrum of compound **1**.

Figure S9. Partial ^{1}H–^{1}H COSY (DMSO-*d*$_6$) spectrum of compound **1**.

Figure S10. HMBC (DMSO-*d*$_6$) spectrum of compound **1**.

Figure S11. Partial HMBC (DMSO-*d*$_6$) spectrum of compound **1**.

Figure S12. Partial HMBC (DMSO-*d*$_6$) spectrum of compound **1**.

Figure S13. Partial HMBC (DMSO-*d*$_6$) spectrum of compound **1**.

Figure S14. Marfey's analysis of the amino acids in **1** on UPLC-MS.

Figure S15. HRESIMS spectrum of compound **1**.

Figure S16. ESI MS^2 spectrum of compound **1**.

Figure S17. The reported centrosymmetric cyclopeptides **3**–**14** from nature.

^{1}H- and ^{13}C NMR data of compound **2**.

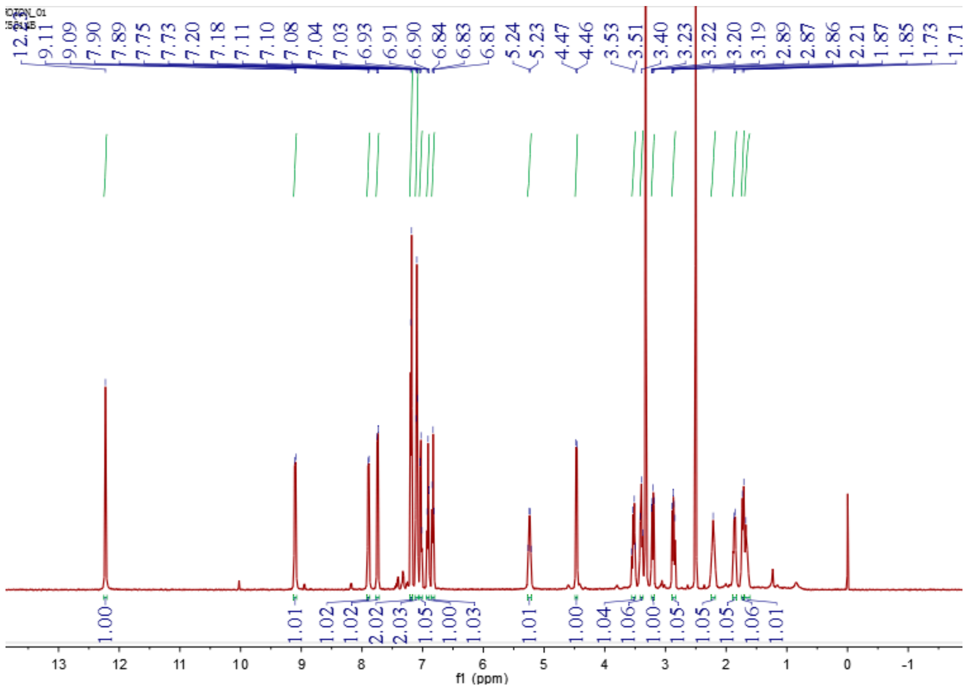

Figure S1. ^{1}H NMR (500 MHz, DMSO-d_6) spectrum of compound **1**.

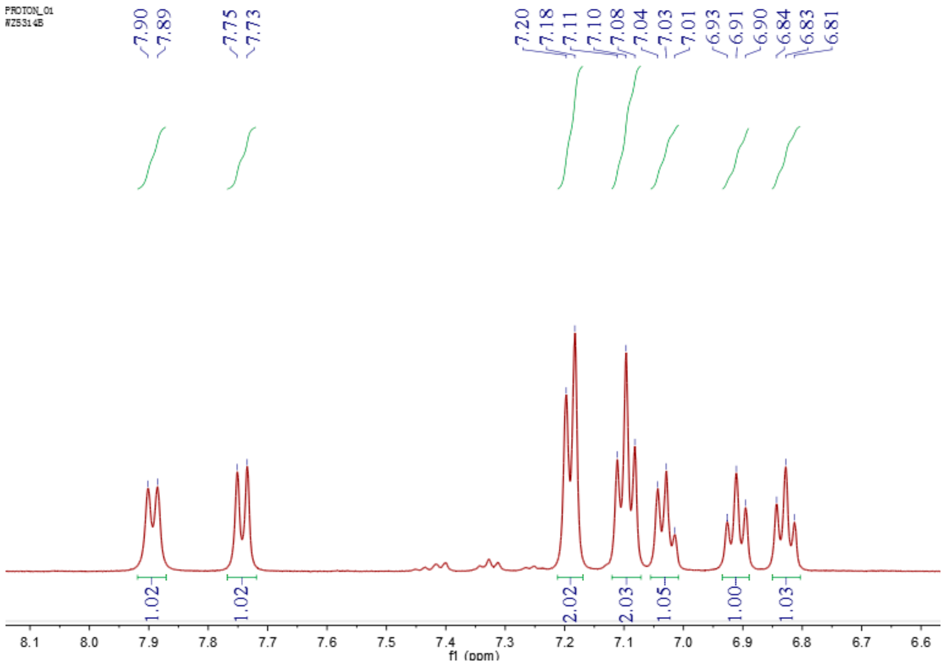

Figure S2. Partial ^{1}H NMR (500 MHz, DMSO-d_6) spectrum of compound **1**.

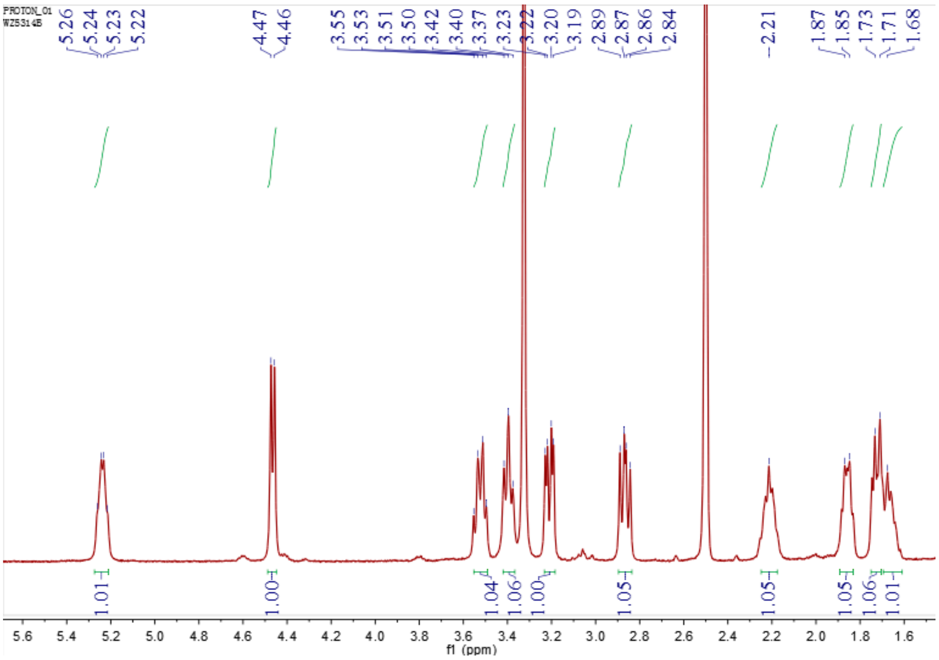

Figure S3. Partial ^{1}H NMR (500 MHz, DMSO-d_6) spectrum of compound **1**.

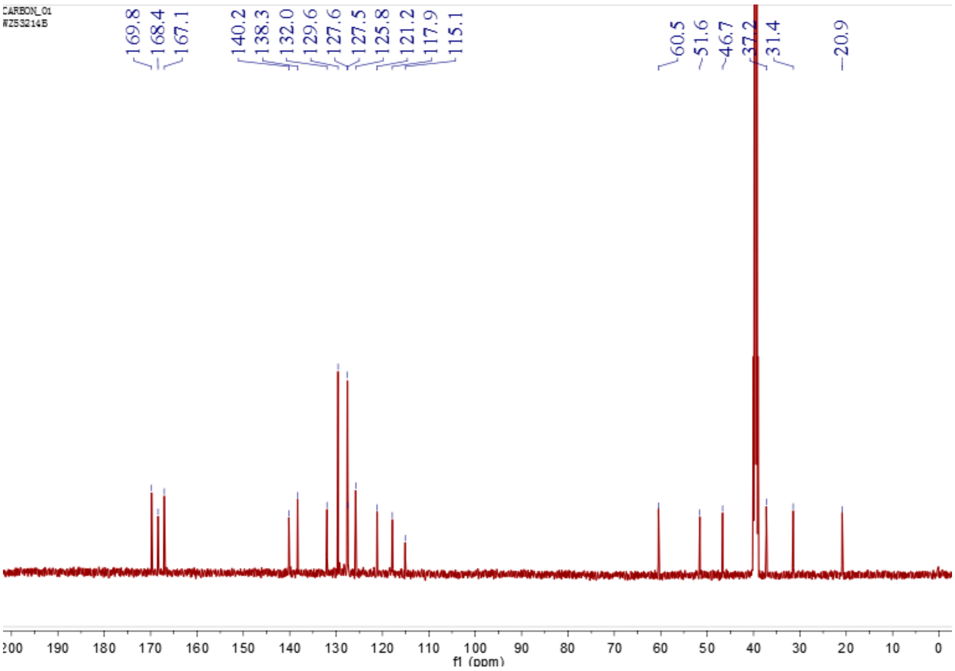

Figure S4. ^{13}C NMR (125 MHz, DMSO-d_6) spectrum of compound **1**.

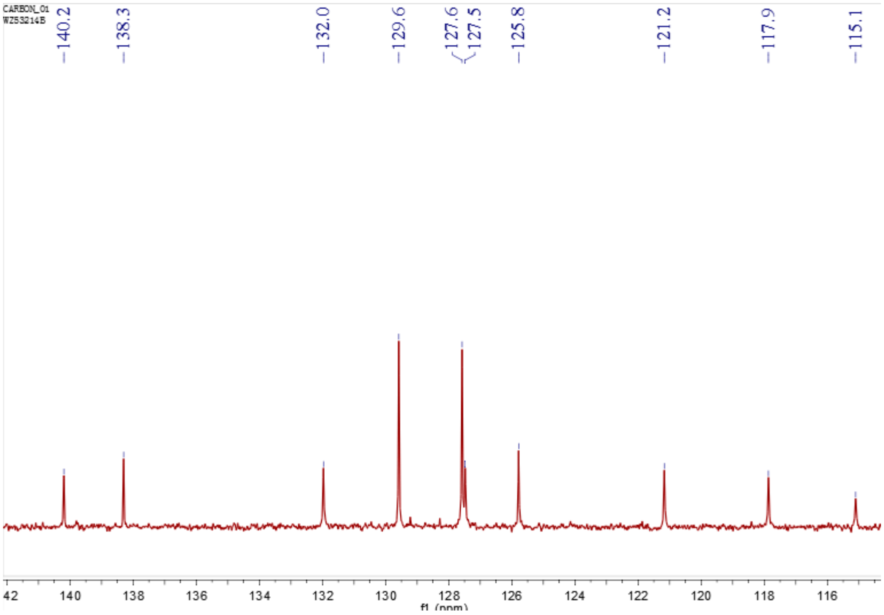

Figure S5. Partial ^{13}C NMR (125 MHz, DMSO-d_6) spectrum of compound **1**.

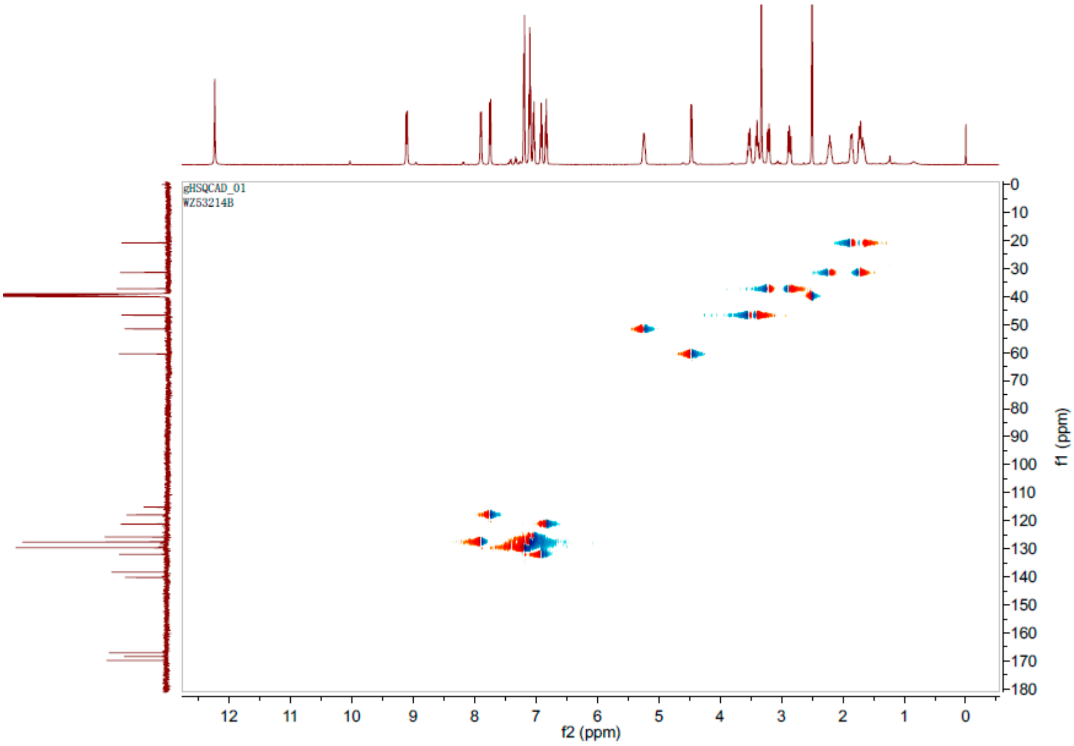

Figure S6. HMQC (DMSO-d_6) spectrum of compound **1**.

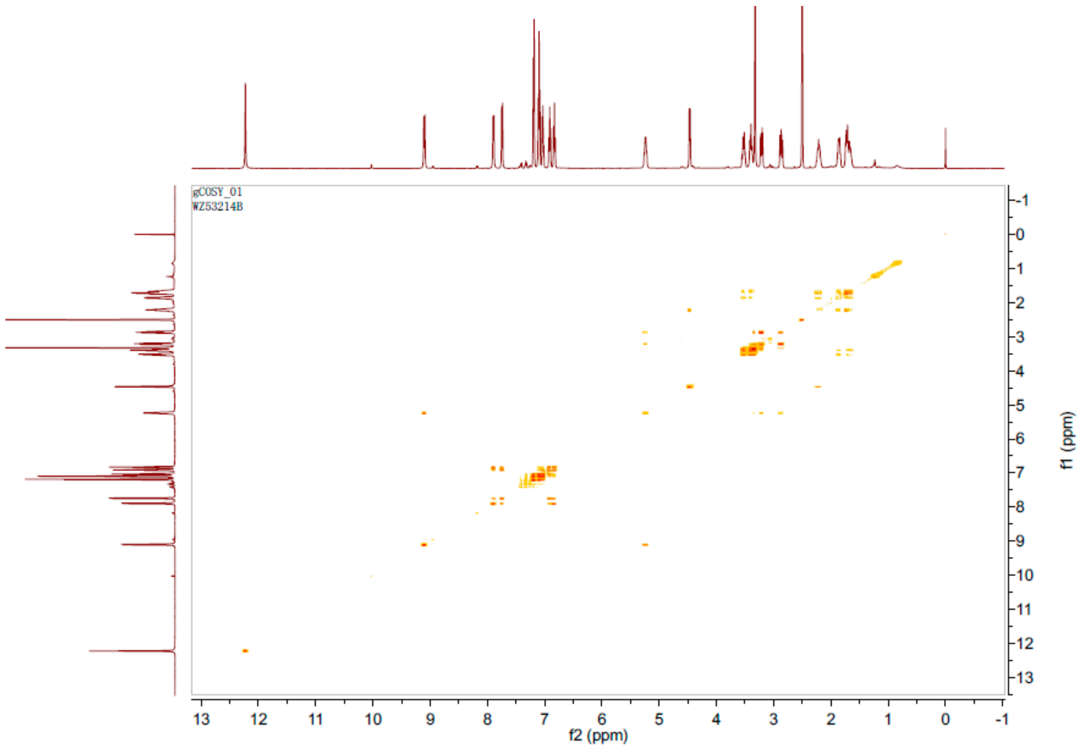

Figure S7. ^1H–^1H COSY (DMSO-d_6) spectrum of compound **1**.

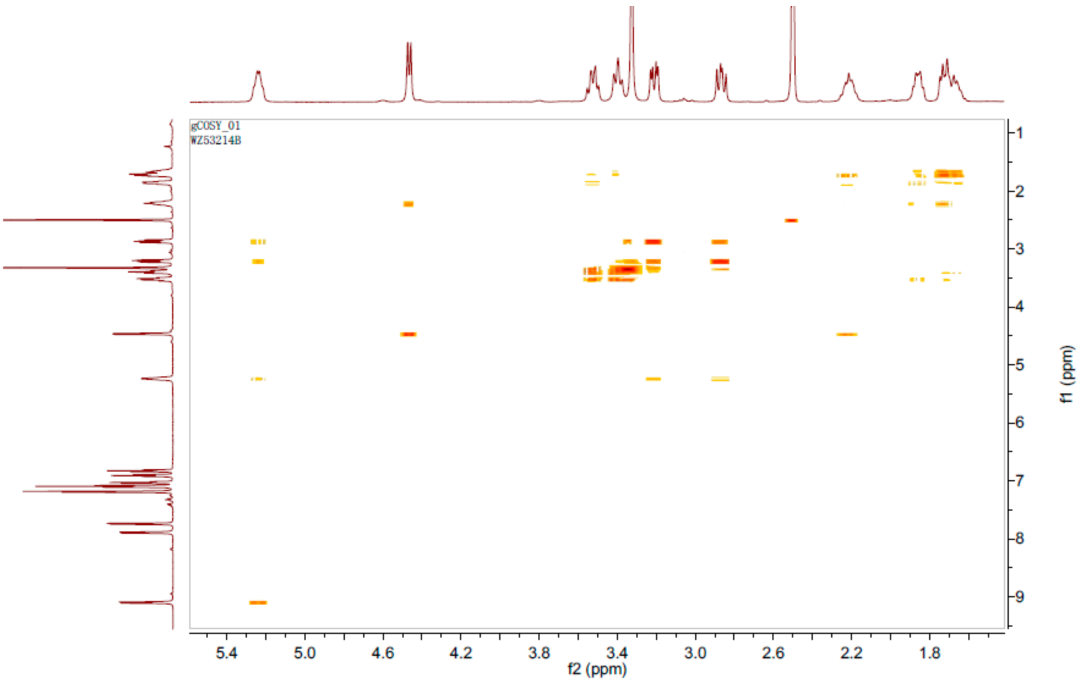

Figure S8. Partial ^1H–^1H COSY (DMSO-d_6) spectrum of compound **1**.

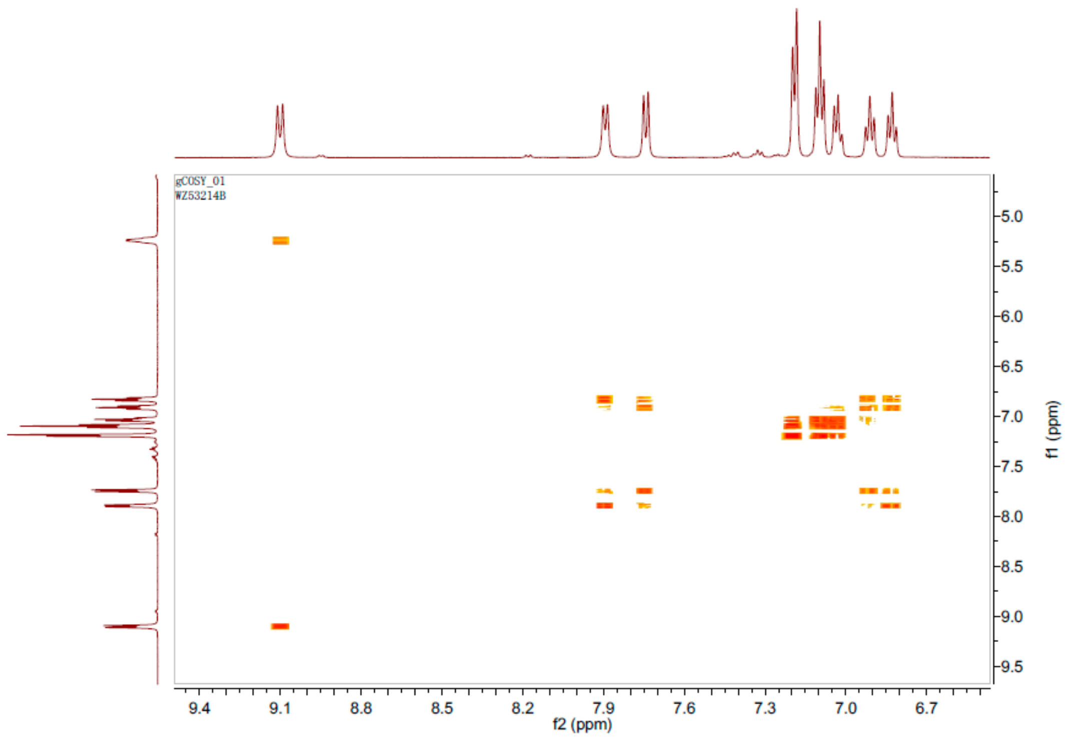

Figure S9. Partial ^{1}H–^{1}H COSY (DMSO-d_6) spectrum of compound **1**.

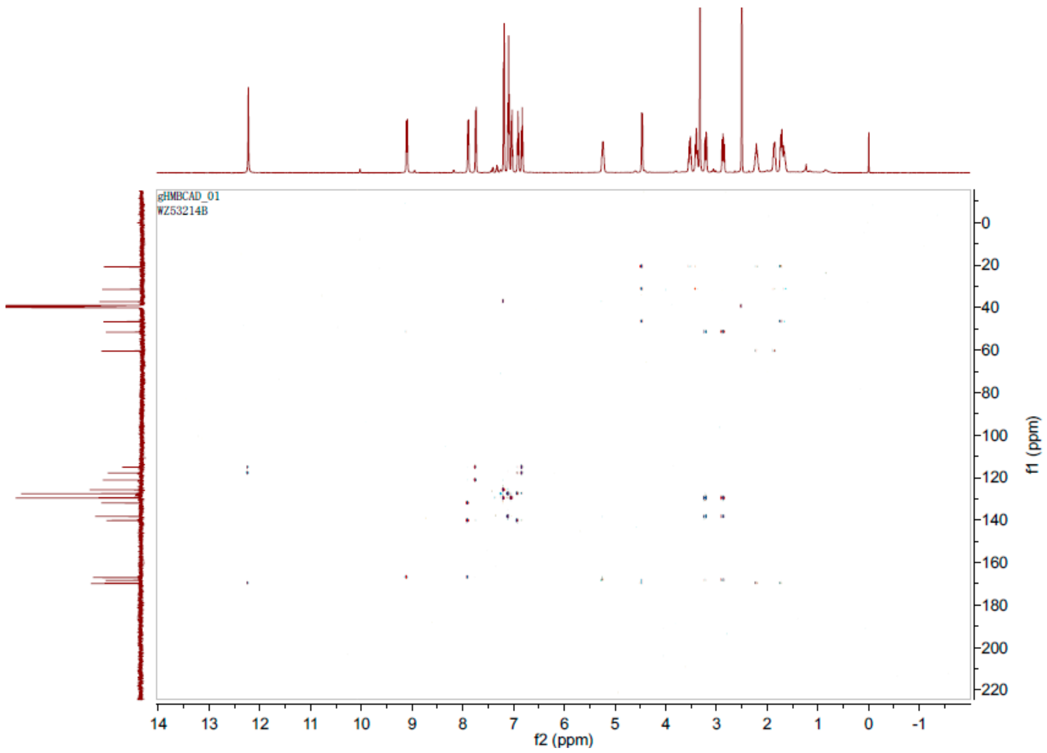

Figure S10. HMBC (DMSO-d_6) spectrum of compound **1**.

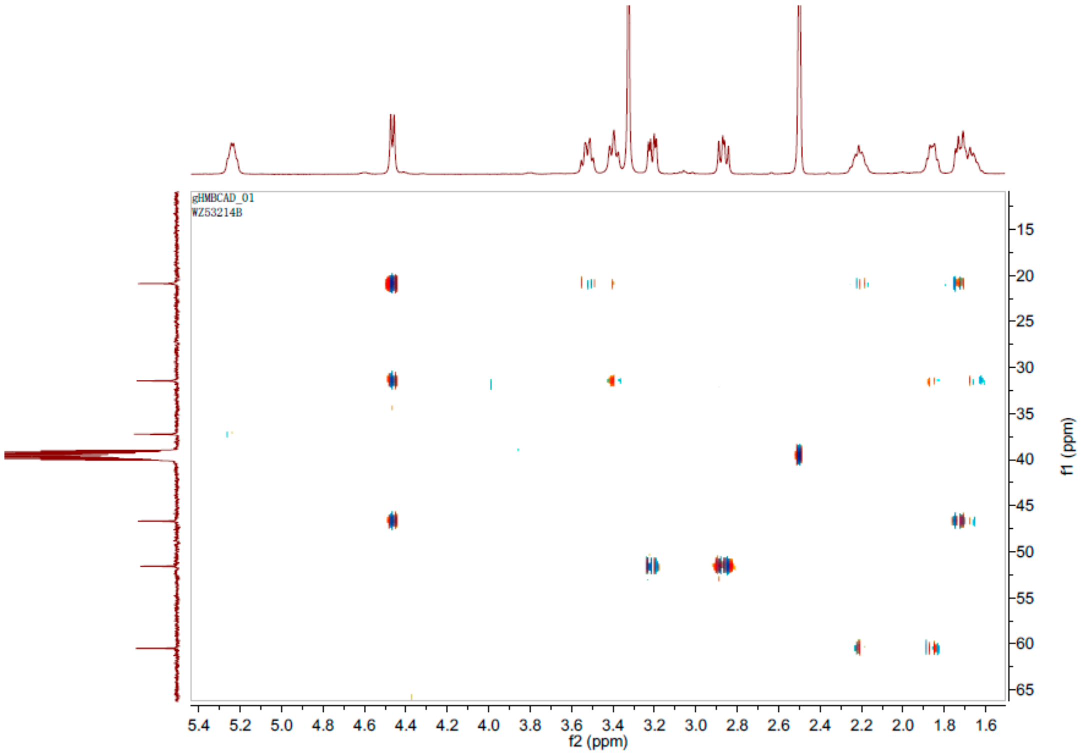

Figure S11. Partial HMBC (DMSO-d_6) spectrum of compound **1**.

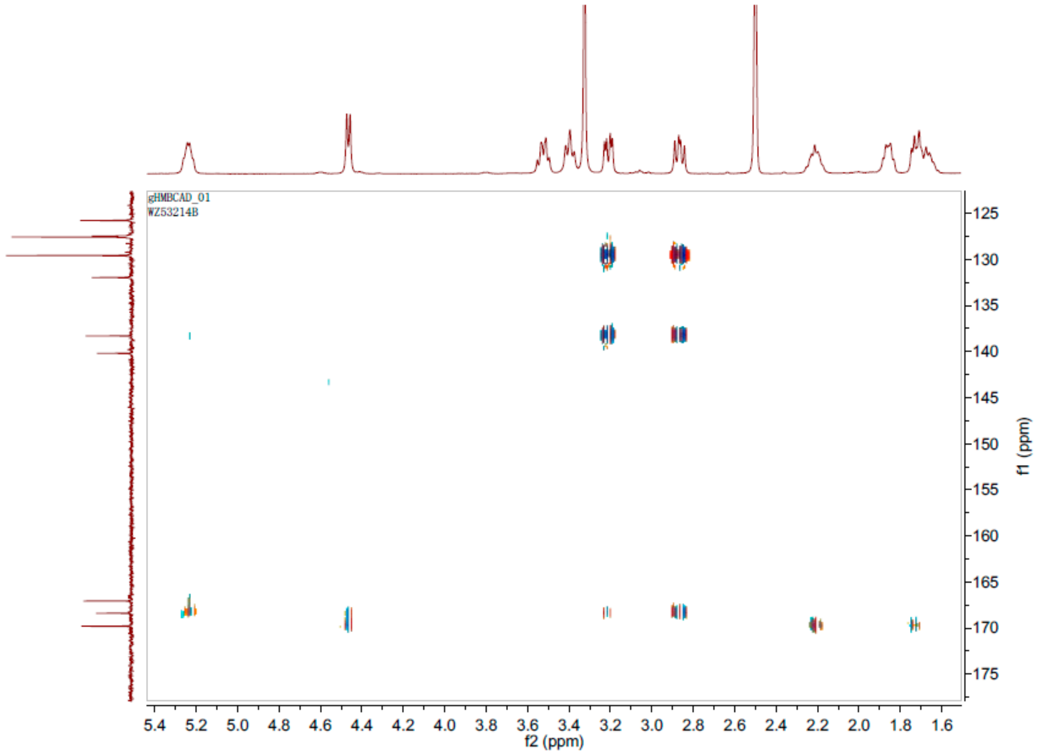

Figure S12. Partial HMBC (DMSO-d_6) spectrum of compound **1**.

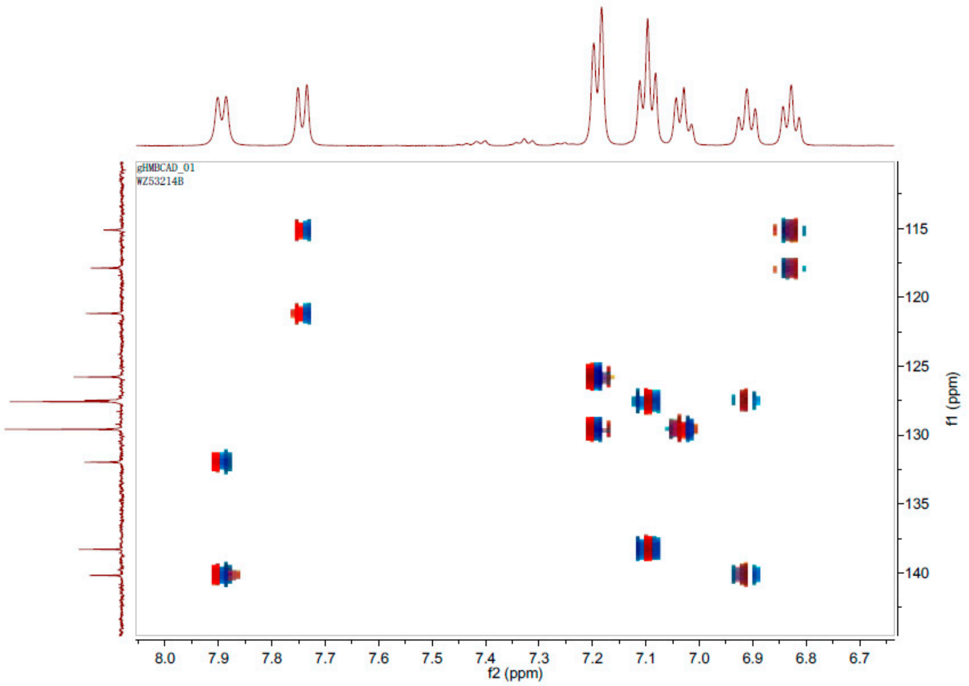

Figure S13. Partial HMBC (DMSO-d_6) spectrum of compound **1**.

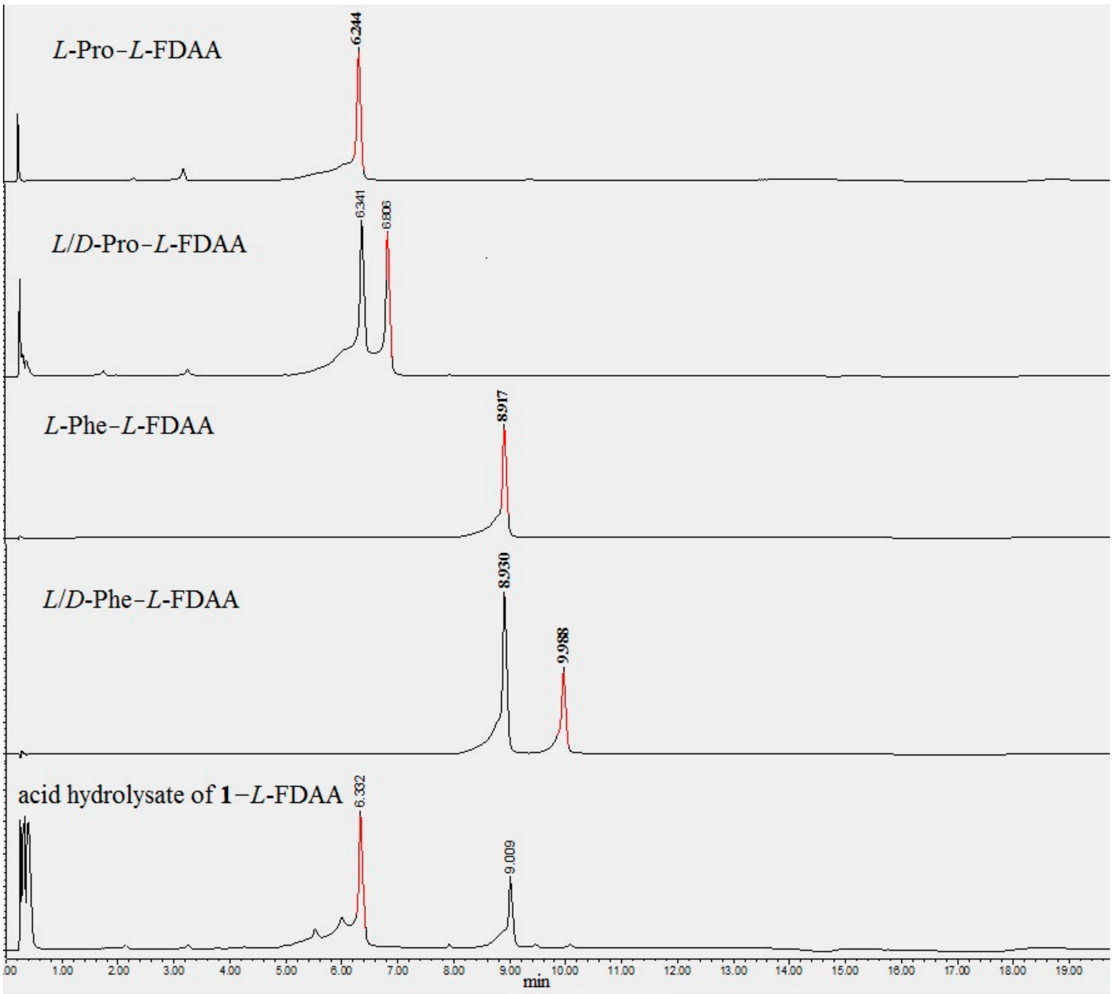

Figure S14. Marfey's analysis of the amino acids in **1** on UPLC-MS

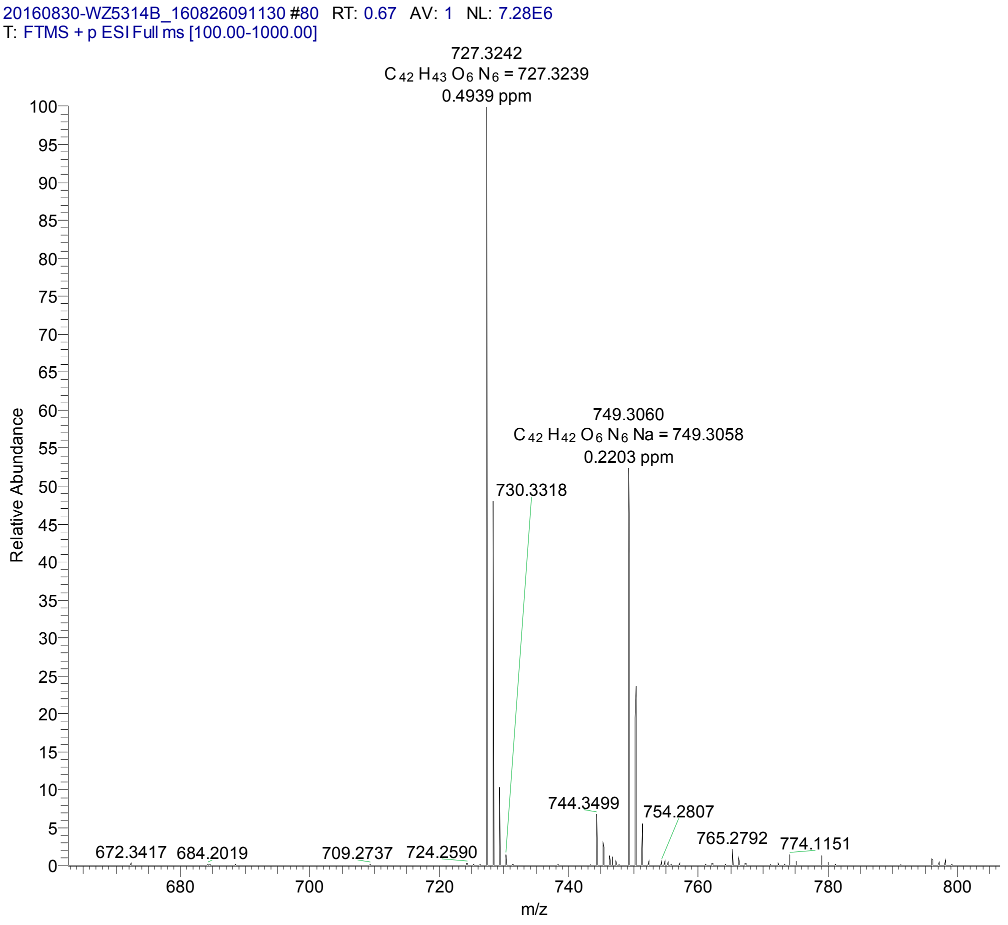

Figure S15. HRESIMS spectrum of compound **1**.

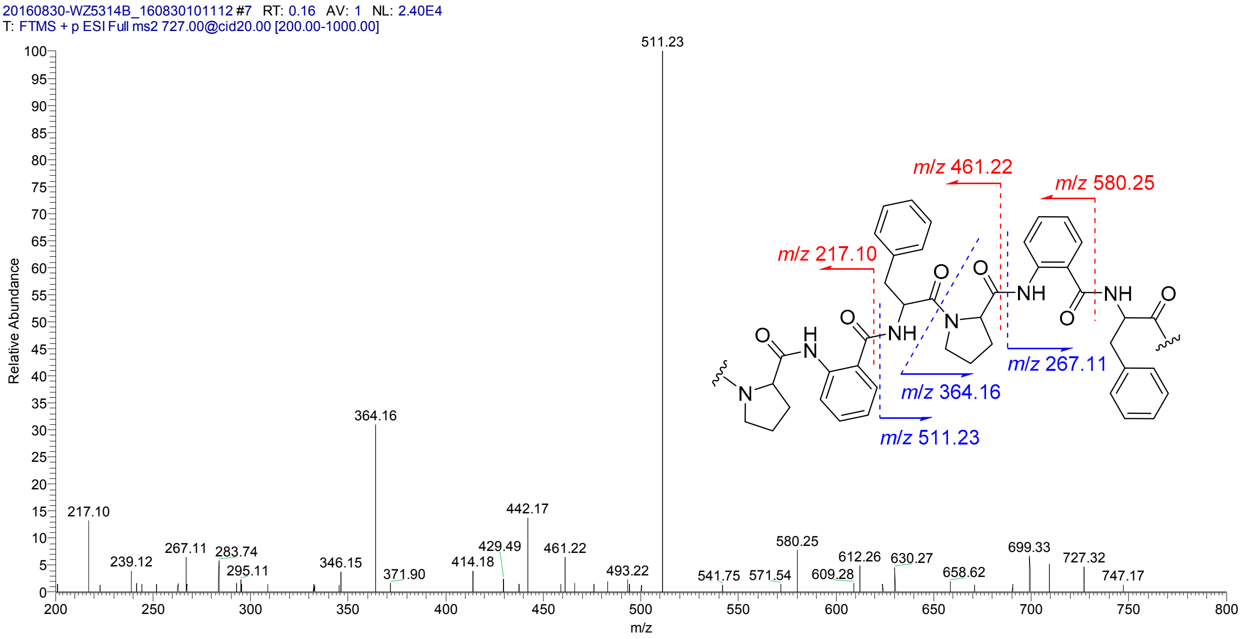

Figure S16. ESI MS^2 spectrum of compound **1**.

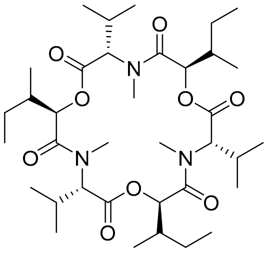
Enniatin A (**3**)

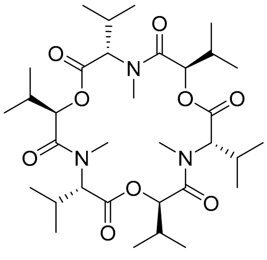
Enniatin B (**4**)

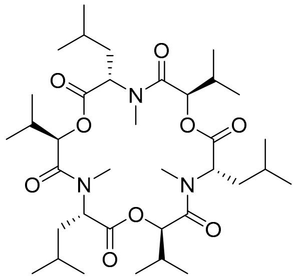
Enniatin C (**5**)

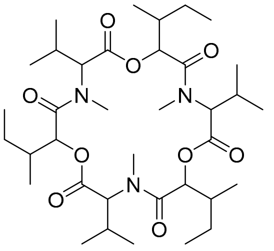
Enniatin MK1688 (**6**)

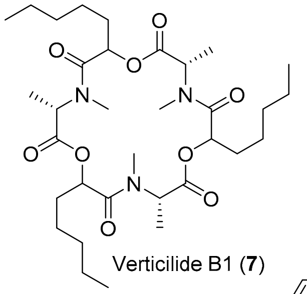
Verticilide B1 (**7**)

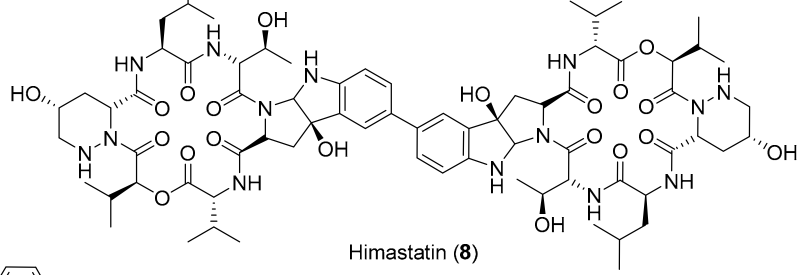
Himastatin (**8**)

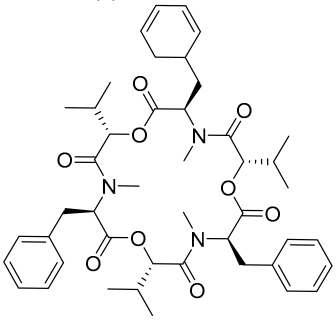
Beauvericin (**9**)

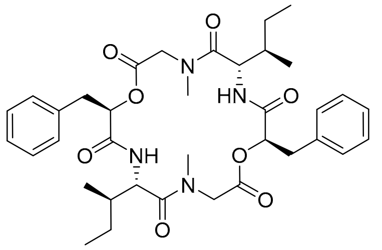
Hirsutellide A (**10**)

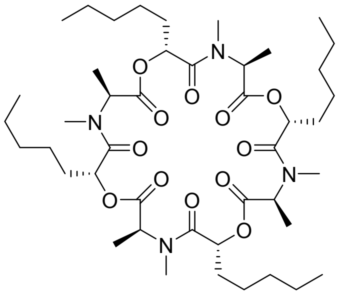
Verticilide A1 (**11**)

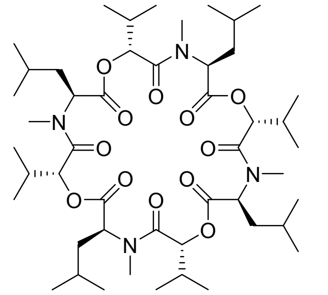
Bassianolide (**12**)

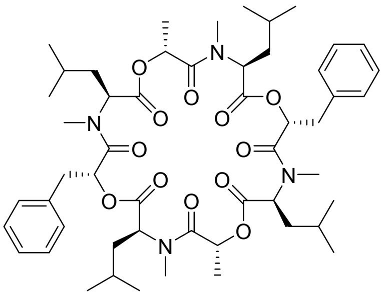
PF1022A (**13**)

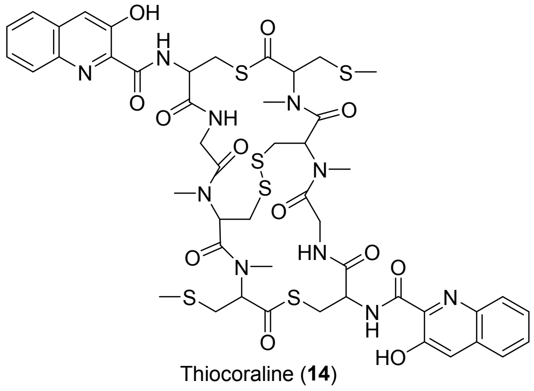
Thiocoraline (**14**)

Figure S17. The reported centrosymmetric cyclopeptides **3**–**14** from nature.

Asperphenamate (2): white powder; ^{1}H NMR (500 MHz, $CDCl_3$, J in Hz) δ_H 7.70 (2H, d, J = 7.5 Hz), 7.66 (2H, d, J = 7.5 Hz), 7.50 (1H, d, J = 7.4 Hz), 7.43 (1H, d, J = 7.4 Hz), 7.39 (2H, d, J = 7.7 Hz), 7.35−7.25 (6H, overlapped), 7.25−7.15 (6H, overlapped), 6.66 (1H, d, J = 8.4 Hz, NH), 6.57 (1H, d, J = 6.4 Hz, NH), 4.92 (1H, q, J = 6.4 Hz, H-2'), 4.62 (1H, dddd, J = 8.4, 6.4, 4.2, 3.1 Hz, H-2), 4.54 (1H, dd, J = 11.3, 3.1 Hz, Ha-1), 4.04 (1H, dd, J = 11.3, 4.2 Hz, Hb-1), 3.29 (1H, dd, J = 13.9, 6.4 Hz, Ha-3'), 3.21 (1H, dd, J = 13.9, 7.0 Hz, Hb-3'), 3.00 (1H, dd, J = 13.6, 6.4 Hz, Ha-3), 2.89 (1H, dd, J = 13.6, 8.4 Hz, Hb-3); ^{13}C NMR (125 MHz, $CDCl_3$) δ_C 172.0 (C, C-1'), 167.6 (C, C-10), 167.3 (C, C-10'), 137.3 (C, C-4), 135.9 (C, C-4'), 134.4 (C, C-11), 133.5 (C, C-11'), 132.2 (CH, C-14), 131.5 (CH, C-14'), 129.4 (CH, C-5/9), 129.3 (CH, C-5'/9'), 129.0 (CH, C-13/15), 128.8 (CH, C-6'/8'), 128.8 (CH, C-6/8), 128.6 (CH, C-13'/15'), 127.5 (CH, C-7'), 127.3 (CH, C-12'/16'), 127.2 (CH, C-12/16), 127.0(CH, C-7), 65.6 (CH_2, C-1), 54.6 (CH, C-2'), 50.4 (CH, C-2), 37.7 (CH_2, C-3'), 37.4 (CH_2, C-3). ESI MS m/z 507 $[M + H]^+$, 529 $[M + Na]^+$.
